# Supplementary material for: Elicitation of Neutralizing Antibody Responses to HIV-1 Immunization with Nanoparticle Vaccine Platforms
Source: Viruses. 2021 Jul 2;13(7):1296. doi: 10.3390/v13071296 (PMC8310022; doi:10.3390/v13071296)
Supplement: Supplementary file 1 [file viruses-13-01296-s001.zip › viruses-1267780-supplementary.pdf]

viruses

MDPI

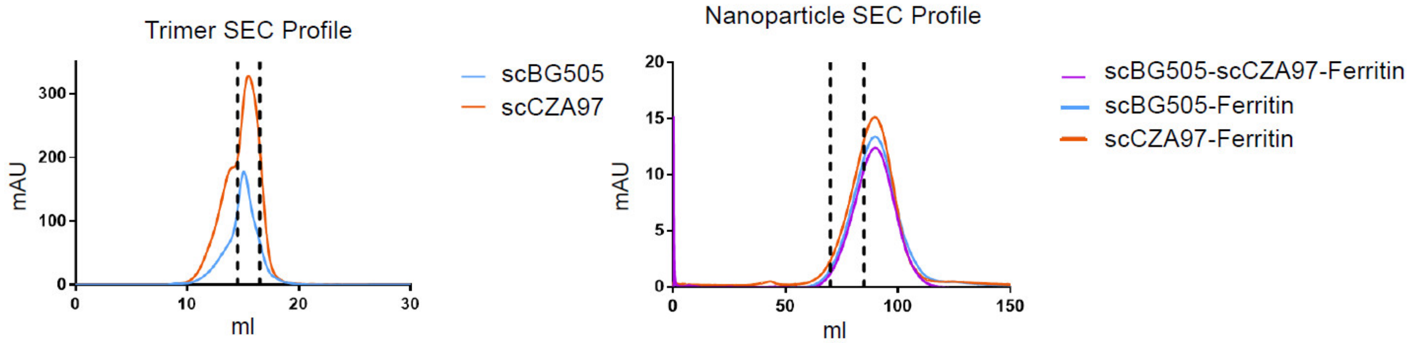

Figure S1. Size-Exclusion Chromatography Profiles of Trimer and Nanoparticle Immunogens. Soluble gp140 trimer immunogens were purified on a Superdex 200 Increase 10/300 GL, and nanoparticle immunogens were purified on 16/60 Sephacryl s500 or Superose 6 columns. Dashed lines denote selected fractions.

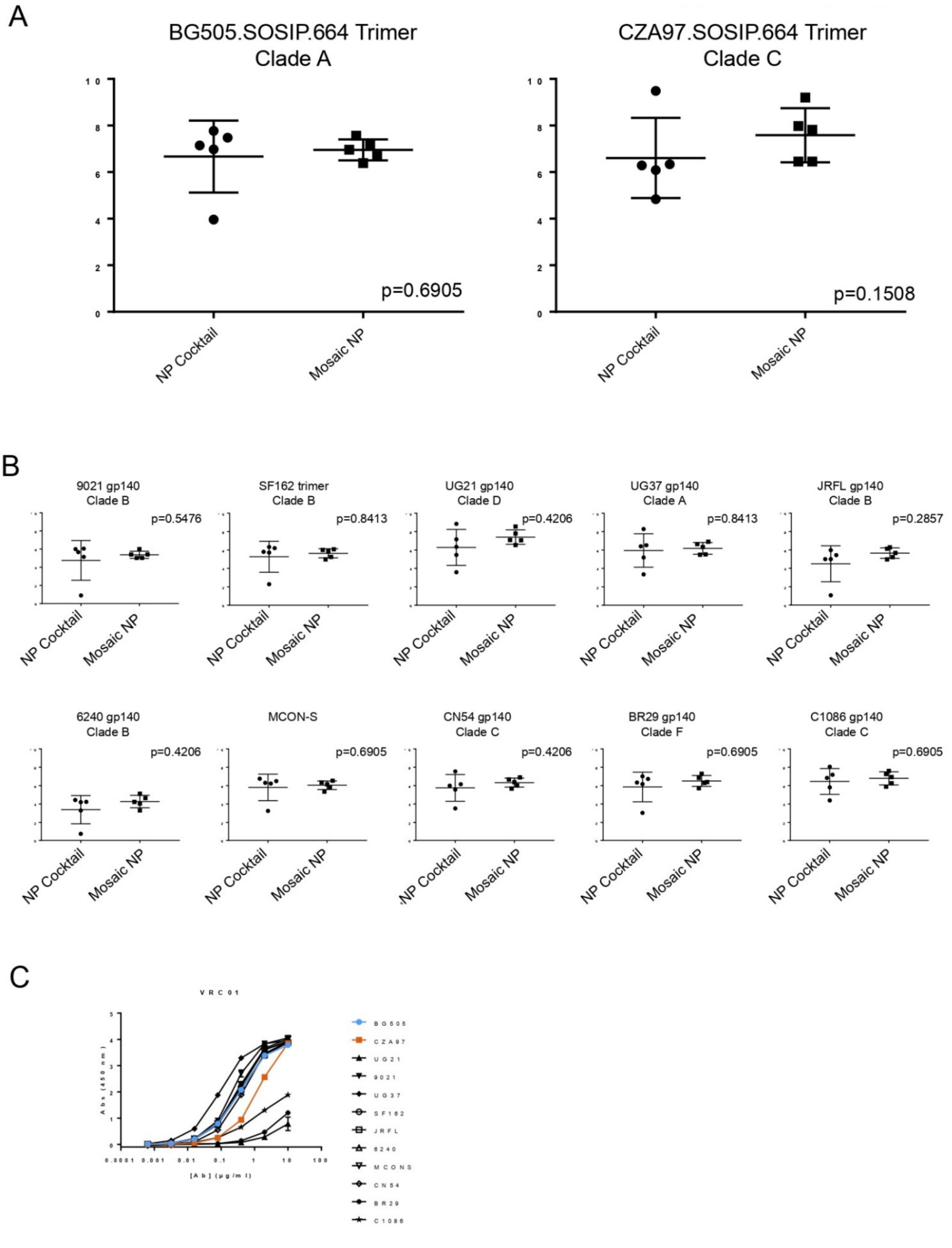

Figure S2. Comparison of AUC values from ELISA Binding Curves of Guinea Pig Serum Against HIV-1 Env Proteins. (**A**) AUC values for vaccine-matched Env trimers plotted from ELISA binding curves in Figure 4B**.** (**B**) AUC values for gp140 Env proteins plotted from ELISA binding curves in Figure 4C. Reported are arithmetic mean and SD p-values from Mann-Whitney U tests. (**C**) Binding for positive control antibody VRC01 against HIV-1 antigens.
